# Giancarlo Comi: a legacy in neurology and multiple sclerosis research

**DOI:** 10.1055/s-0045-1806830

**Published:** 2025-04-27

**Authors:** Maria Fernanda Mendes

**Affiliations:** 1 Santa Casa de São Paulo School of Medical Sciences, São Paulo SP, Brazil.

## BIOGRAPHICAL OVERVIEW

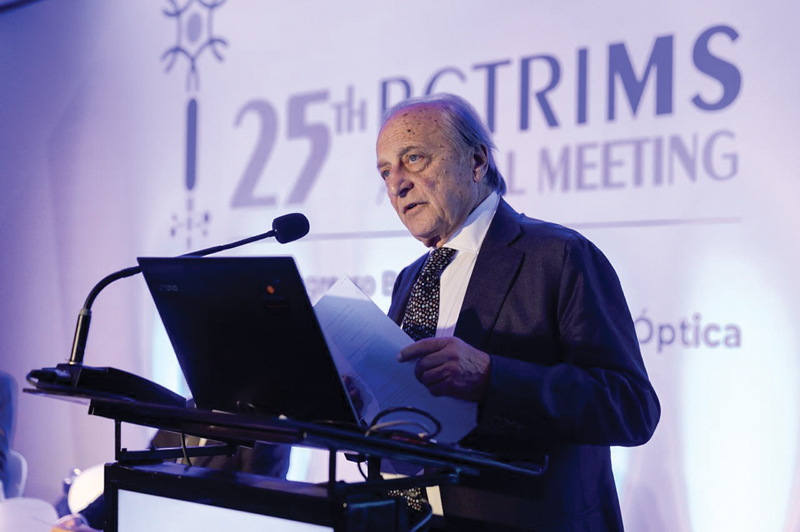



Giancarlo Comi was born in Carvico, Italy, on December 15, 1947, and passed away on November 26, 2024. He was one of those physicians who made a lasting impact on multiple sclerosis (MS) research and treatment. He authored over 1,000 scientific papers published in prestigious international journals, edited several scientific books, and had an h-index of 100. He organized and spoke at over 600 scientific conferences worldwide, both nationally and internationally. He served on the executive boards of various scientific associations and on the editorial boards of *Clinical Investigation* , *European Journal of Neurology* , and *Multiple Sclerosis* , among others. 1 He was also a member of the Editorial Advisory Board of the journal *Arquivos de Neuro-Psiquiatria: Neurology and Neuroscience.* As a pioneer in the early diagnosis and treatment of MS, he championed the involvement of people with MS in research globally, encouraging studies and advancing MS knowledge in many countries, always with great kindness and generosity.

## ACADEMIC AND PROFESSIONAL MILESTONES

Giancarlo Comi earned his medical degree in 1973 and Neurology certification in 1977 at Milan University. He joined the Department of Neurology at San Raffaele in 1974 and later held key roles, including Professor of Neurology, Department Chairman, and Director of the Institute of Experimental Neurology (Istituto di Neurologia Sperimentale – INSPE, in Italian) at Vita-Salute San Raffaele University. From the mid-1980s, he pioneered new MS approaches, introducing magnetic resonance imaging (MRI) and advanced diagnostics while emphasizing early diagnosis. In Italy, he helped secure the first MRI machine dedicated to MS research in collaboration with the Italian MS Society. 2

Throughout his career, he held prominent positions, serving as President of the European Neurology Society, the Italian Society of Clinical Neurophysiology, and the Italian Society of Neurology. From the early stages of his professional journey, Professor Comi was actively engaged with two of the most influential organizations dedicated to MS: the European Committee for Treatment and Research in Multiple Sclerosis (ECTRIMS) and the European Charcot Foundation, which he co-founded and was and supported ardently later. These roles allowed him to disseminate knowledge and connect people, not only in Italy and European community but also across the global scientific and medical landscape. Professor Comi received numerous international awards, including the prestigious Charcot Award in 2015, which acknowledged him as one of the world's leading experts on MS. 3 He was actively involved in steering committees and advisory boards for numerous international clinical trials, mainly in MS, and served on the executive boards of various scientific associations.

His strong commitment to collaboration earned him honorary memberships in several institutions around the world. He was also recognized as an honorary member of the Brazilian Committee for Treatment and Research in Multiple Sclerosis (BCTRIMS) for his unwavering engagement, support of scientific initiatives, and efforts to integrate Brazilian researchers into the global scientific community. Consistently active in academia, he served as President of the European Charcot Foundation, Executive Council Member of ECTRIMS from 2003 to 2007, becoming an honorary member in 2014. 4 He recently served as Honorary Professor at Vita-Salute University, Chairman of the Scientific Committee of the Human Brains Prada Foundation, and Director of the MS Center at Casa di Cura Igea in Milan.

## FOCUS ON PEOPLE WITH MS

Central to Professor Comi's philosophy was his unwavering commitment to improving the lives of individuals with MS. 2 From the early days of his career, he demonstrated this dedication, presenting a study advocating a multidisciplinary approach to MS treatment at the inaugural ECTRIMS Congress in 1985. He was a pioneer in promoting early intervention in MS while highlighting the benefits of rehabilitation. In his home country, he played a pivotal role in encouraging the establishment of MS treatment centers, advocating for patients' rights and quality of life. In 2018, he led the launch of the Multiple Sclerosis Care Unit initiative, a structured, multidisciplinary model for the global management of MS that brings together patients, nurses, and specialists. 5

Professor Comi emphasized patient-centered research models, leading initiatives like the MULTI-ACT consortium, to unite stakeholders: patients, patient organizations, academic institutions, government agencies, and technology partners. He also supported the creation of the International Progressive MS Alliance, 6 an initiative that brings together various community stakeholders to develop effective treatments for progressive MS. For many years, he served on the the Italian MS Foundation (Fondazione Italiana Sclerosi Multipla –FISM, in Italian) board of directors, shaping impactful research projects and guiding MS studies to improve patient outcomes.

Professor Comi also championed the Patient-Reported Outcome for Multiple Sclerosis (PROMS) initiative at the 35th ECTRIMS Congress, emphasizing the importance of patient-reported outcomes in MS research and care. The PROMS initiative is jointly led by the European Charcot Foundation (ECF) and the Multiple Sclerosis International Federation (MSIF), with the Italian MS Society (Associazone Italiana Sclerosi Multipla – AISM, in Italian) as the lead agency representing the global MSIF movement. 7 Just days before his passing, he chaired a PROMS meeting, reiterating his belief that people with MS must guide research priorities to address their real needs.

## MENTOR AND COLLABORATOR

Professor Comi was recognized for his exceptional ability to communicate complex topics with clarity, engaging and discussing intricate aspects of research with honesty and simplicity. Above all, he was a dedicated scientist who never lost his passion for teaching, research, and inspiring new generations of scientists. He always believed in global collaboration, working with researchers worldwide, including partnerships with Brazil.

Professor Comi's first visit to Brazil, in 2000, marked the beginning of a deep connection with the country's culture and its neuroimmunology community. He returned regularly, participating in numerous events and fostering collaboration. He frequently spoke at BCTRIMS congresses, always sharing his expertise and inspiring young neuroimmunologists to pursue research in Brazil. At the 25th BCTRIMS congress, he expressed his admiration for the event's growth and the advancements in Brazilian neuroimmunology.

Beyond his groundbreaking contributions to MS research, Giancarlo Comi will be remembered as a compassionate physician, an inspiring educator, and a visionary leader. His relentless pursuit of knowledge and his dedication to improving the lives of people with MS leave an indelible mark on the field of neurology and beyond. He was a cherished friend to many Brazilian neuroimmunologists, leaving a legacy of excellence and inspiration that will continue to guide future generations.
